# Synzymes: The Future of Modern Enzyme Engineering

**DOI:** 10.1007/s12010-025-05305-1

**Published:** 2025-07-05

**Authors:** Ahmet Alperen Palabiyik

**Affiliations:** Department of Nursing, Faculty of Health Sciences, Ardahan University, Ardahan, Turkey

**Keywords:** Synzymes, Synthetic enzymes, Enzyme engineering, Biocatalysis

## Abstract

Synzymes, synthetic mimics of natural enzymes, have emerged as a promising frontier in modern biocatalysis due to their enhanced stability, adaptability, and catalytic performance. Unlike natural enzymes, synzymes are engineered to function under extreme physicochemical conditions, making them suitable for a broad range of applications in biomedicine, industrial biotechnology, and environmental remediation. This review provides a comprehensive overview of the structural principles, functional mechanisms, and real-world applications of synzymes. Particular attention is given to their role in targeted drug delivery, biosensing, green manufacturing, and pollutant degradation. Furthermore, the integration of artificial intelligence and high-throughput screening technologies has accelerated synzyme design, enabling more efficient and cost-effective development pipelines. By evaluating both the innovations and current limitations in synzyme research, this article outlines the growing potential of synthetic enzymes as next-generation tools for sustainable and precision-driven solutions.

## Introduction

Enzymes are essential for life and are considered the first biocatalysts on Earth [1]. They are highly efficient biocatalysts due to their fast reaction rates, near maximum possible speed, high-rate accelerations (up to 10^20^ compared to uncatalyzed reaction), use of relatively mild reaction conditions (typically pH 6–8 and temperatures between 20 and 45 °C, centered around physiological conditions such as neutral pH and 37 °C), and high chemo-, regio-, and stereoselectivity [2, 3].

The global enzyme market was valued at approximately USD 7.1 billion in 2023 and is projected to reach USD 10.2 billion by 2028, driven by growing demand in sectors such as pharmaceuticals, food and beverage, biofuels, diagnostics, and waste treatment [4, 5]. Synzymes, or synthetic enzymes, are synthetic catalysts designed to replicate the biochemical functions of natural enzymes while providing enhanced stability, adaptability, and efficiency across diverse environmental conditions [6]. These artificial enzymes have emerged as a powerful tool in modern enzymology by addressing several limitations associated with naturally occurring enzymes [7]. One of the main drivers behind the development of enzymes is the need for highly efficient and robust catalysts that can function under extreme pH, temperature, and solvent conditions that often limit the application of natural enzymes [8]. One of the ultimate goals of modern enzyme engineering is also the rational design of stable enzymes with specifically designed properties, such as specific (acting only on certain substrates), regiospecific (preferred sites within the substrate molecule), or stereospecific (synthesis of compounds with defined chiral configurations) [9].

The concept of synthetic enzymes has its roots in early work in biomimetic catalysis, where researchers attempted to design molecular structures that mimic the active sites of enzymes [10]. Over the past few decades, advances in nanotechnology, supramolecular chemistry (which focuses on molecular recognition and non-covalent interactions such as hydrogen bonding and host–guest chemistry), and computational modeling have propelled the field forward, leading to the development of highly efficient synthetic catalysts [11]. These catalysts are designed to stabilize transition states, improve reaction kinetics, and exhibit substrate specificity comparable to or even superior to natural enzymes [12].

Unlike natural enzymes, which are typically derived from living organisms and can lose function under harsh conditions, synzymes are chemically synthesized and engineered to retain catalytic activity across a wide range of environmental conditions [13, 14]. This allows their application in non-biological systems such as industrial reactors, polluted environments, and synthetic biological circuits [15]. This distinction between natural and synthetic enzymes is critical in determining their practical utility. One of the most promising areas of enzyme research is their application in medicine, particularly in drug delivery, cancer treatment, and antimicrobial therapies [16]. Synthetic peroxidases and oxidases have demonstrated remarkable efficacy in neutralizing oxidative stress, a critical factor in many diseases [17]. Besides oxidative stress, synzymes have also been explored in biosensing, gene editing, and neuroprotection models, extending their relevance in emerging therapeutic areas [18]. In addition, the ability of synzymes to function in complex biological environments has paved the way for their integration into diagnostic tools, biosensors, and targeted therapeutic systems [19].

In industrial biotechnology, synzymes offer a sustainable alternative to traditional catalysts, enabling greener chemical processes with reduced waste and energy consumption. Their use in pharmaceutical synthesis, polymerization, and biofuel production has been widely investigated, and their potential to improve process efficiency and cost-effectiveness has been demonstrated [20]. Moreover, environmental applications of synzymes have gained momentum, particularly in bioremediation, pollutant degradation, and carbon capture technologies. Synthetic enzyme systems designed to degrade persistent organic pollutants and heavy metals hold promise for addressing global environmental challenges [21].

A brief historical timeline summarizing key milestones in synzyme research illustrates the field’s progression from early biomimetic concepts to sophisticated therapeutic and industrial applications. This chronological development is summarized in Table 1, which outlines major breakthroughs in synzyme research from the 1970 s to the present.

**Table 1:** Key milestones in the historical development of synzyme research

Year	Milestone
1970s	First biomimetic catalytic molecules synthesized
1996	Term “synzyme” first introduced in enzymology literature
2000	First DNAzyme developed, capable of site-specific catalysis
2003	Synthetic peroxidase mimics show biological activity
2015–2020	Integration of nanomaterials and metal–organic frameworks into synzyme scaffolds
2020–2024	AI-assisted enzyme design and large-scale biomedical applications begin to emerge

## Structural and Functional Principles of Synzymes

These synthetic enzymes utilize host–guest chemistry, a supramolecular interaction in which a host molecule non-covalently binds a specific guest molecule, along with hydrogen bonding, van der Waals interactions, and hydrophobic effects to stabilize substrate binding and facilitate biochemical transformations [19].

Advances in supramolecular chemistry have enabled the design of artificial active sites that selectively bind target molecules with high affinity and specificity, enhancing catalytic efficiency and substrate specificity [21].

One of the key principles of synzyme catalysis is the stabilization of the transition state in chemical reactions [22]. By reducing the activation energy, synzymes accelerate reaction rates similarly to natural enzymes [22, 23]. Various strategies have been employed to achieve this, including metal-based catalysis, organic framework-supported reactions, and the use of molecular scaffolds that provide structural rigidity to active sites [24, 25]. Metal–organic frameworks (MOFs) and metalloenzyme mimics have been particularly successful in developing stable and efficient synthetic catalysts [26]. These systems incorporate metal ions such as zinc, copper, and iron into engineered frameworks, allowing precise control over reaction environments and catalytic pathways [27].

**Fig. 1:**
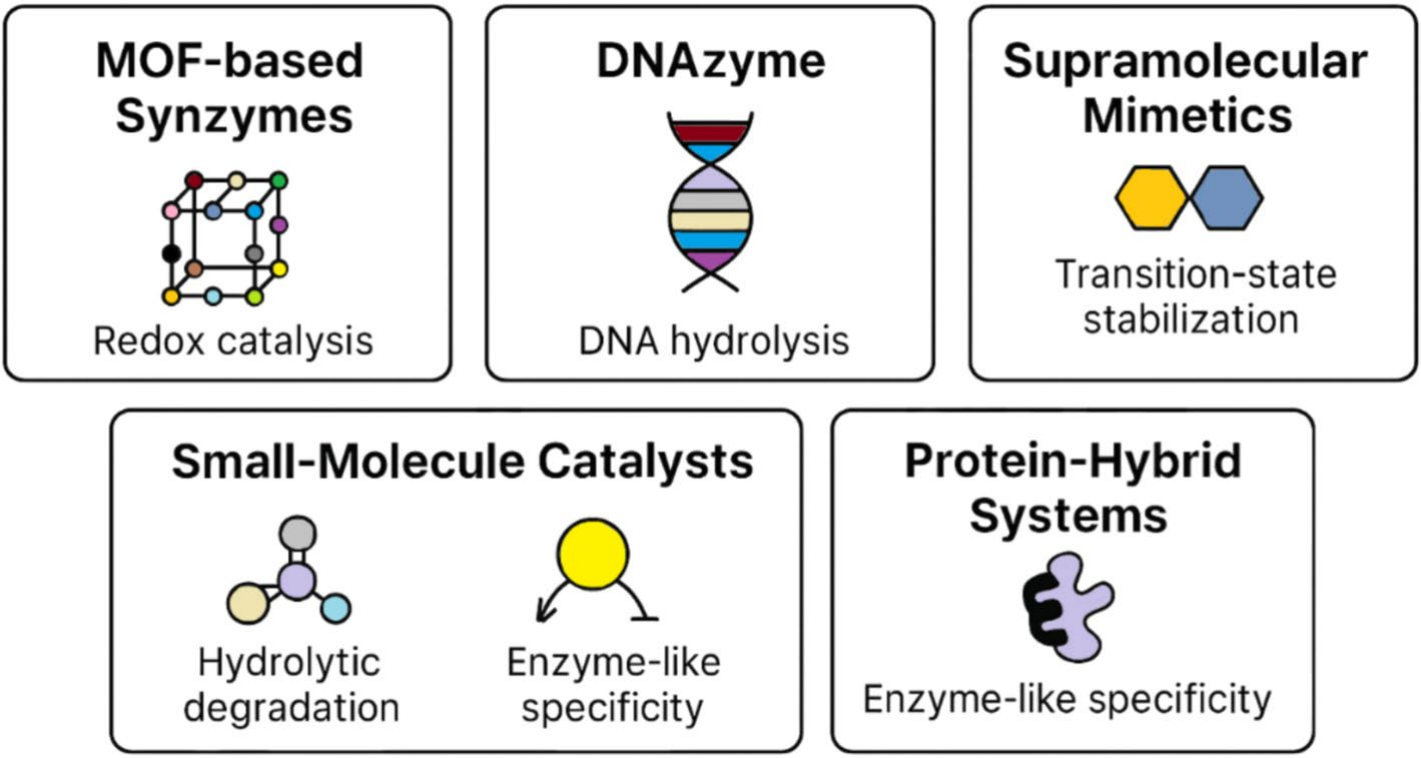
Diversity of synzyme scaffolds and catalytic architectures

Different types of synthetic enzymes have been developed to meet the demands of diverse biochemical and industrial applications [19]. MOFs provide porous materials that act as enzyme mimics, offering high surface areas and tunable catalytic properties [28, 29]. Supramolecular enzyme mimetics leverage self-assembled molecular architectures to replicate the active sites of natural enzymes, improving stability and functional versatility [30]. DNA-based artificial enzymes utilize the programmability of nucleic acids to perform highly specific biochemical reactions, whereas small molecule catalysts offer efficient alternatives for oxidation–reduction reactions, hydrolysis, and other catalytic transformations [31, 32]. Synzymes can be constructed using a wide range of molecular platforms, each tailored to specific catalytic functions and environmental conditions. These include MOFs, DNA-based structures, and hybrid protein systems, all of which offer unique advantages in stability and substrate specificity (Fig. 1).

The figure categorizes key scaffold types utilized in synzyme engineering, including MOF-based constructs, DNAzymes, supramolecular mimetics, small-molecule catalysts, and protein-hybrid systems. Each architecture supports distinct catalytic mechanisms such as redox catalysis, DNA hydrolysis, transition-state stabilization, and hydrolytic degradation. The figure highlights the structural and mechanistic diversity that defines modern synthetic enzyme systems. Created by the author for illustrative purposes.

As research continues to expand, synzymes have been increasingly applied in biomedicine, industry, and environmental science, offering novel solutions where traditional biocatalysts fall short. This versatility stems from their engineered stability and adaptability to harsh operational conditions.

For example, MOF-based nanozymes have shown high peroxidase-like catalytic efficiency and remarkable environmental robustness, making them valuable in biosensing and therapeutic applications [33]. Similarly, RNA-cleaving DNAzymes exhibit high substrate specificity and turnover numbers in the range of 1–5 min^−1^, enabling their use in gene regulation and diagnostics [34].

These distinguishing features are summarized in Table 2, which compares natural enzymes and synzymes in terms of structure, function, and practical applicability.

**Table 2:** Comparative characteristics of natural and synthetic enzymes grouped by structure, function, and application parameters

Category	Natural enzymes	Synthetic enzymes
Structure	Derived from biological macromolecules (proteins, ribozymes)	Chemically engineered frameworks (MOFs, DNAzymes, small molecules)
Stability	Sensitive to environmental factors (pH, temperature, solvents)	High stability across broad pH, temperature, and solvent ranges
Substrate specificity	Naturally evolved, high specificity	Tunable specificity via design and selection
Catalytic efficiency	High under optimal physiological conditions	Comparable or superior in non-natural conditions
Customization	Limited by evolutionary constraints	Readily modified for target applications
Production method	Extracted via fermentation or cell culture	Synthesized chemically or via nanofabrication
Production cost	Often high (bioprocessing, purification)	Potentially lower; scalable and reproducible
Applications	Medicine, industry, biotechnology, environment	Medicine, biosensing, industrial catalysis, environmental remediation

## Synthetic Enzyme Creation and Isolation

The creation of synthetic enzymes begins with the rational design of catalytic sites that mimic natural enzyme function [35]. Using computational modeling and molecular docking techniques, researchers predict optimal active site configurations that enhance substrate binding and transition state stabilization [36]. This is followed by the chemical synthesis of enzyme-mimetic structures, often using nanomaterials, MOFs, or supramolecular assemblies that exhibit catalytic properties [37].

Recent advancements have seen the integration of artificial intelligence (AI) in enzyme engineering. AI techniques, such as machine learning algorithms, have been employed to analyze complex datasets, predict molecular interactions, and accelerate the design of enzymes with enhanced functionality. For instance, AI-driven molecular modeling has facilitated the prediction of protein structures and interactions, expediting the development of synthetic enzymes with desired properties [38].

Once synthesized, synthetic enzymes undergo extensive characterization to confirm their structural integrity and functional efficacy [39]. Techniques such as X-ray crystallography, nuclear magnetic resonance (NMR) spectroscopy, and electron microscopy are employed to analyze the molecular architecture of synzymes [40]. Functional assays, including kinetic studies and substrate specificity tests, are performed to evaluate their catalytic efficiency compared to natural enzymes [41].

A typical characterization workflow includes:

1. Structural validation via spectroscopic and imaging techniques
2. Purity analysis by chromatography and mass spectrometry
3. Performance testing under various conditions to benchmark stability and reactivity

**Fig. 2:**
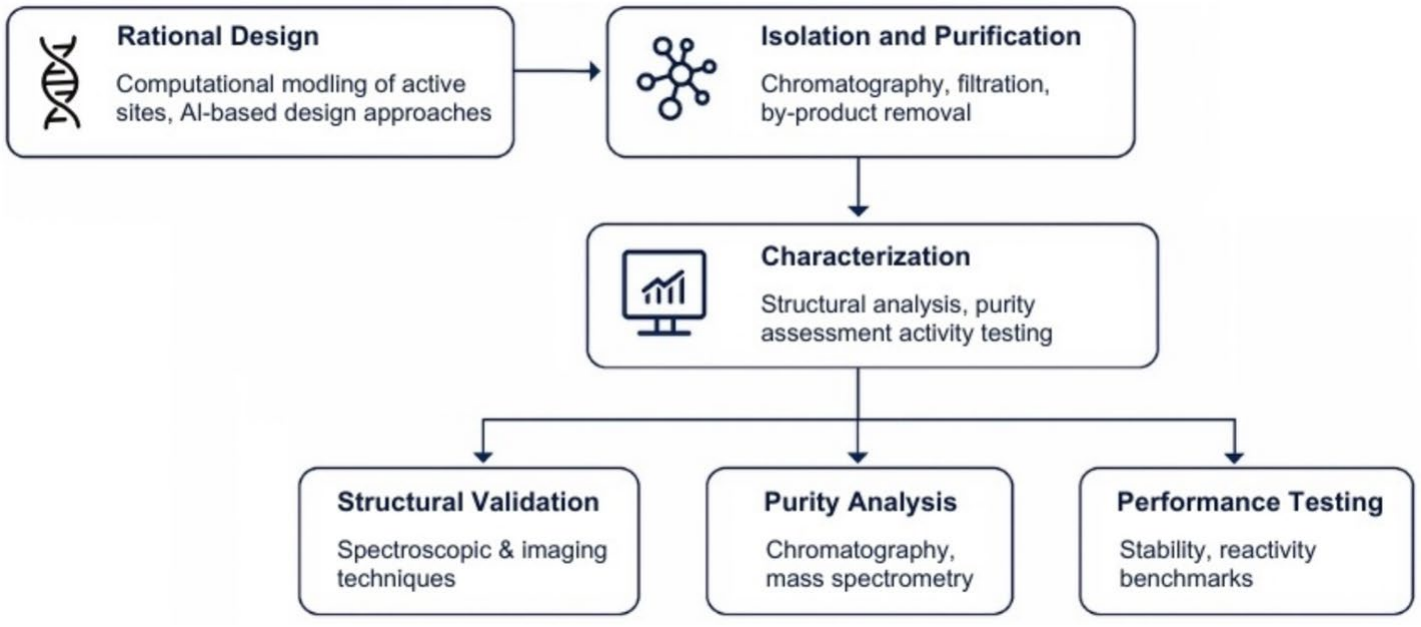
Workflow of synzyme development and characterization

Figure 2 illustrates the integrated workflow of synzyme development and characterization, encompassing computational design, chemical synthesis, purification, and multi-level validation strategies.

The schematic outlines the key stages of synzyme engineering, including rational design, isolation and purification, and characterization through structural validation, purity analysis, and performance testing. Created by the author for illustrative purposes.

Isolation and purification of synthetic enzymes involve chromatographic techniques such as high-performance liquid chromatography (HPLC) and gel filtration chromatography to separate active synzyme molecules from unwanted by-products [42]. Additionally, mass spectrometry is used to validate the molecular weight and purity of the synthesized enzymes [43]. These steps ensure that the final product maintains high catalytic activity and stability under desired reaction conditions [44].

However, the synthetic process often results in the formation of non-functional analogs, residual reagents, or unreacted precursors. These by-products can interfere with catalytic performance and pose environmental risks if improperly handled. Strategies such as phase-transfer catalysis, green solvent systems, and post-synthesis purification protocols are actively being developed to mitigate these concerns [45, 46].

One of the major challenges in synthetic enzyme development is achieving high specificity while maintaining stability in diverse environments [47, 48]. Researchers are exploring hybrid approaches that integrate synthetic and biological elements, such as incorporating protein scaffolds into artificial catalysts to enhance biocompatibility and efficiency. This bio-hybrid strategy has shown promise in extending the functional range of synzymes within physiological systems, particularly in drug delivery and intracellular sensing. Table 3 compares representative natural enzymes and their synthetic counterparts, highlighting differences in structure, synthesis method, catalytic properties, and application domain.

**Table 3:** Comparative features of natural enzymes and synzymes

Natural enzyme	Synthetic counterpart	Structure	Synthetic strategy	Catalytic activity (*k*_cat_)	Stability	Improved stability in synzyme	Application
Horseradish peroxidase (HRP)	MOF-based peroxidase mimic	Heme protein	MOF embedding	~ 2500 s^−1^	Sensitive to pH/temp	High (broad pH/temp)	Biosensors, assays
Ribonuclease A (RNase A)	DNAzyme	RNA-cleaving protein	DNA sequence catalysis	~ 1–5 min^−1^	Moderate in vitro	Programmable, stable	RNA cleavage, biosensing
Carbonic anhydrase	Zn (II)-based artificial enzyme	Zinc metalloenzyme	Metal coordination complex	~ 10^6^ s^−1^	Moderate	Stable across solvents	CO_2_ capture, biocatalysis
Lipase	Supramolecular lipase mimic	Protein hydrolase	Self-assembled micellar catalyst	~ 1,000 s^−1^	Limited in organic solvents	Enhanced organic tolerance	Detergents, biofuels

Table 4 summarizes selected studies on synthetic enzyme strategies, detailing catalytic performance and application areas across biomedical and industrial contexts.

**Table 4:** Selected studies on synzyme strategies and performance

Enzyme type	Synzyme strategy	Performance metric	Application
Peroxidase	MOF-based catalyst	*k*_cat_/*K*_M_ = 2.1 × 10^5^ M^−1^ s^−1^	Glucose sensing
DNAzyme	Catalytic DNA	*k*_cat_ = 1–5 min^−1^	Gene regulation
Carbonic anhydrase	Metal-complex mimic	CO_2_ hydration rate enhanced	CO_2_ fixation
Lipase mimic	Micellar system	Ester hydrolysis in organic phase	Detergent formulation

## Applications of Synzymes

Synzymes have broad applications across biomedicine, industrial biotechnology, environmental science, and diagnostics [49]. In biomedicine, they are increasingly used in cancer therapy, where catalytic drug delivery systems enable precise targeting and controlled drug release [50, 51]. They also serve as antibacterial and antiviral agents, acting as synthetic oxidases or peroxidase-like enzymes that generate reactive oxygen species (ROS) to combat pathogens [52]. Another crucial application in healthcare is the regulation of oxidative stress, where synzymes mimic natural peroxidase activity to neutralize harmful free radicals [53]. For instance, a copper-MOF-based synzyme has demonstrated peroxidase-like activity with a catalytic efficiency (*k*_cat_/*K*_M_) of 2.1 × 10^5^ M^−1^ s^−1^, significantly outperforming natural horseradish peroxidase under similar conditions [33].

In industrial biotechnology, synzymes enhance the efficiency of biocatalytic processes in pharmaceutical synthesis, replacing natural enzymes that are sensitive to environmental factors [54]. Their role in biofuel production is also significant, as they facilitate the conversion of complex biomaterials into usable energy sources [55]. Furthermore, synzymes are used in catalytic polymerization processes, aiding in the synthesis of biodegradable plastics and other polymeric materials [56].

**Fig. 3:**
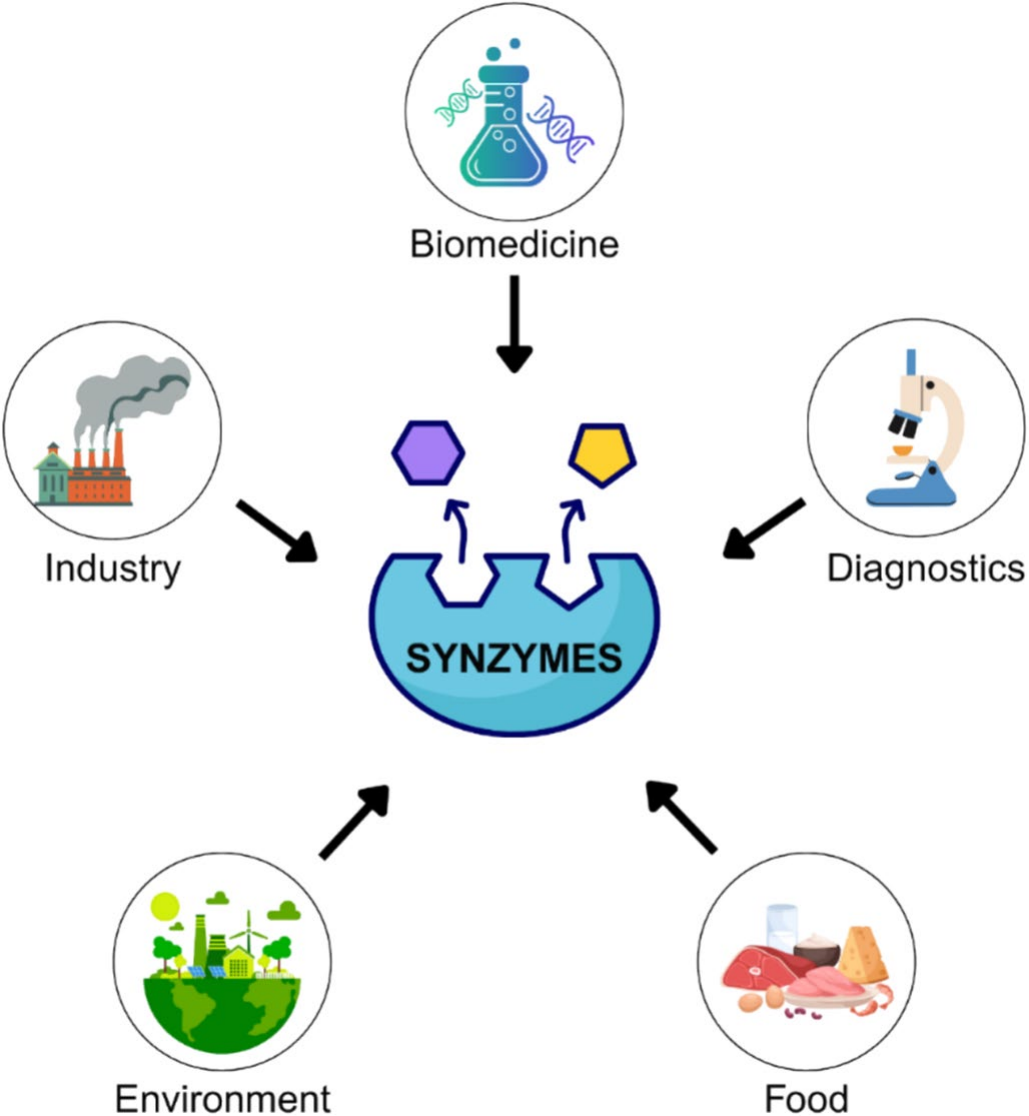
Sector-based applications of synzymes. This schematic illustrates the major sectors where synzymes have demonstrated significant application potential, including biomedicine, diagnostics, food processing, industry, and environmental remediation. Each domain benefits from the tailored catalytic activity and specificity of synzyme platforms. Created by the author for illustrative purposes

Environmental applications of synzymes are crucial for pollution control and sustainability [57]. They contribute to the biodegradation of harmful pollutants by breaking down toxic substances into less harmful compounds [58]. Synzymes are also implemented in water purification systems, where they help detoxify contaminants and improve water quality [59]. Another innovative application is their role in carbon dioxide capture and conversion, where they mimic natural carbonic anhydrase enzymes to facilitate CO_2_ sequestration and utilization [60]. A zinc-coordinated artificial carbonic anhydrase synzyme was shown to accelerate CO_2_ hydration reactions by up to 500-fold, supporting its use in sustainable carbon capture technologies [61] (Fig. 3).

Emerging applications of synzymes include their integration into wearable biosensors and gene therapy platforms. Their high substrate specificity, stability, and ability to operate under physiological conditions make them ideal candidates for smart, real-time diagnostic systems embedded in skin patches, clothing, or implantable devices. Moreover, synthetic nucleic acid-based enzymes (e.g., DNAzymes) are being explored as gene regulators in programmable therapeutics, offering new directions in precision medicine [62].

Diagnostics and sensing technologies benefit from synzyme-based biosensors, which provide rapid and highly specific detection of biomolecules [63]. These biosensors are utilized in medical diagnostics, environmental monitoring, and food safety testing. Additionally, synzymes are integrated into colorimetric and fluorescence-based detection systems, offering precise and real-time monitoring capabilities for various analytes. The versatility of synzymes across these fields highlights their growing significance in scientific and industrial advancements [64, 65]. A broad overview of synzyme applications across biomedical, industrial, environmental, and diagnostic fields is presented in Table 5, illustrating their versatility and technological relevance in modern science.

**Table 5:** Multisectoral applications of synzymes: key fields, functions, and catalytic examples

Application area	Specific use	Example synzyme type
Biomedical	Targeted drug delivery, cancer therapy, oxidative stress regulation	Peroxidase-mimicking nanozymes
Industrial	Pharmaceutical synthesis, biofuel production, polymerization	Metal–organic framework (MOF) synzymes
Environmental	Wastewater treatment, plastic degradation, CO_2_ capture	Laccases, PETases
Food and beverage	Fermentation optimization, flavor enhancement	Amylases, lipases
Diagnostics	Rapid detection in biosensors	DNA-based synthetic enzymes

### Chemical Synthesis: Design of New Molecules

Modern societies are heavily reliant on functional chemicals, necessitating large-scale production strategies to meet demands across consumer, industrial, and pharmaceutical markets [66]. The sensory attributes taste, smell, and texture of many affordable commodities are optimized using synthetic chemistry and are often delivered in plastic-based packaging, contributing to mounting environmental and public health concerns [67]. As a response, the chemical industry is undergoing a paradigm shift toward more sustainable and environmentally conscious processes, replacing conventional high-energy, toxic, or non-renewable synthetic routes [68].

The development of modern enzyme engineering and protein design has revolutionized the synthesis of new molecules with precise control over reactivity and selectivity [69]. Protein engineers manipulate the amino acid sequences of enzymes, altering their three-dimensional conformation to perform highly efficient, multistep reactions [70]. Directed evolution techniques have expanded the catalytic repertoire of enzymes to include reactions involving non-natural substrates, enabling the generation of novel proteins that bridge the functional space between chemo- and biocatalysts. This synthetic-biological interface has become especially attractive for green chemistry applications and industrial biotransformations [33].

Enzymes are macromolecular catalysts that have evolved to perform highly specific reactions in living systems. They offer catalytic power, substrate control, and product specificity under mild conditions physiological pH, ambient temperatures, and aqueous solvents, making them nontoxic and environmentally benign [71, 72]. Advances in structural biology and mechanistic enzymology over the last decades have significantly enhanced our understanding of enzyme behavior, enabling the rational design of de novo proteins tailored for non-native chemical tasks [73, 74].

One of the first and most influential artificial enzyme systems was demonstrated by Ronald Breslow and Larry Overman in 1970, who synthesized a molecule combining a metal catalytic center with a hydrophobic binding pocket. This construct mimicked esterase activity, marking a milestone in the birth of biomimetic catalysis [75]. Their work laid the foundation for subsequent efforts to build synthetic systems that replicate or even surpass natural enzymatic functions in both aqueous and non-biological environments.

AI has recently emerged as a transformative tool in enzyme design, accelerating the identification and enhancement of synthetic biocatalysts [76]. AI-driven platforms integrate structural databases, molecular dynamics simulations, and predictive learning algorithms to guide the rational generation of catalytic frameworks, significantly reducing the trial-and-error cycle of conventional methods.

**Fig. 4:**
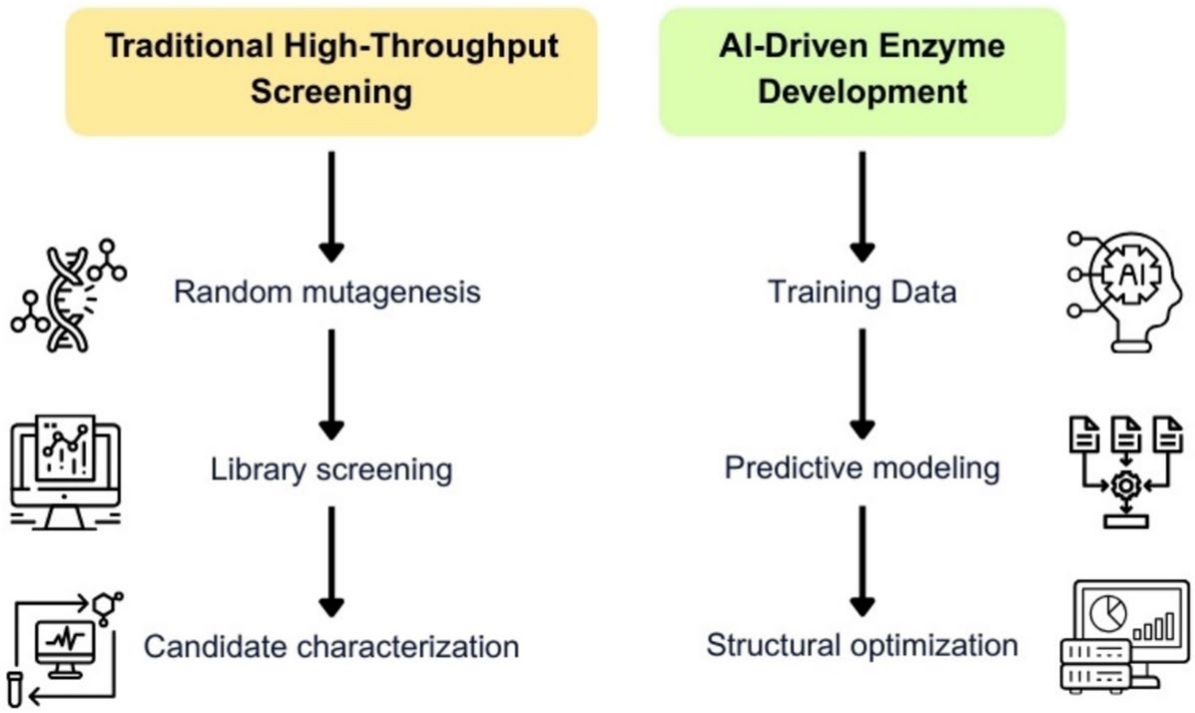
Comparison of traditional high-throughput screening and AI-driven enzyme development approaches. This diagram contrasts classical enzyme discovery workflows based on random mutagenesis and empirical screening with modern AI-guided methods that utilize predictive modeling and structural optimization. AI approaches significantly reduce experimental workload while enhancing specificity and success rates. Created by the author for illustrative purposes

Figure 4 compares conventional enzyme discovery strategies, such as random mutagenesis followed by high-throughput screening, with AI-driven workflows that leverage predictive modeling and structural optimization—highlighting gains in precision, speed, and resource efficiency.

## Latest Technologies in Synzymes Optimization

### Artificial Intelligence and Machine Learning in Enzyme Design

Enzyme catalysts play a pivotal role in both industrial bioprocesses and biological systems; however, their rational design is often limited by the complexity of protein folding, the vast mutational space, and the labor-intensive nature of traditional experimental approaches [76]. AI and machine learning (ML) have emerged as transformative tools that systematically guide enzyme optimization by integrating structural bioinformatics, biochemical data, and mechanistic modeling [77].

Machine learning algorithms are now widely applied to predict enzyme–substrate interactions, design novel catalytic motifs, and explore evolutionary trajectories using large, annotated datasets. These methods enable the creation of both de novo synthetic enzymes and optimized variants of natural enzymes, enhancing efficiency, sustainability, and selectivity in industrial biocatalysis [78].

One of the most significant advances has been the use of deep learning models such as AlphaFold and RoseTTAFold for accurate tertiary structure prediction of enzymes, including artificial constructs. These models leverage massive sequence databases and spatial constraints to predict atomic-level protein folding, accelerating the structural validation of synzymes prior to synthesis. Additionally, graph neural networks (GNNs) are used to represent protein structures as node-edge frameworks, enabling the identification of catalytically active residue networks and predicting the impact of mutations on activity [79].

Unlike conventional scaffold-based enzyme engineering, AI-based workflows bypass empirical limitations by searching sequence-function landscapes through generative design. This allows the exploration of enzyme activities beyond natural capabilities and unlocks novel biotransformation pathways [38]. For example, a recent AI-designed copper-binding synzyme mimicked oxidase activity with enhanced thermal stability and a 3.2-fold increase in *k*_cat_/*K*_M_ over its biological counterpart [61].

Beyond structure prediction, AI enables fully automated high-throughput screening pipelines that can rank thousands of enzyme variants based on predicted binding energy, active site flexibility, and folding robustness. Reinforcement learning strategies have been applied to iteratively refine enzyme sequences toward specific goals such as substrate specificity or solvent tolerance [80].

Despite their potential, AI approaches face limitations such as overfitting due to sparse experimental data, low interpretability of deep models (the “black box” problem), and the challenge of bridging in silico predictions with in vitro validation. These challenges necessitate the integration of wet-lab feedback into AI pipelines to refine models and improve prediction reliability [81].

Deep learning architectures such as convolutional neural networks (CNNs), transformers, and variational autoencoders (VAEs) are also increasingly used to optimize enzyme reaction conditions. These include temperature, pH, cofactor concentrations, and substrate ratios leading to improved yields, reduced byproducts, and expanded industrial relevance of synzymes.

### High-Throughput Screening Techniques

High-throughput screening (HTS) is a cornerstone of modern enzyme engineering, enabling the rapid evaluation of thousands of enzyme variants for desirable traits such as catalytic activity, substrate specificity, and thermostability [82]. In the context of synzymes, synthetic enzymes with engineered catalytic functions, HTS is particularly valuable for optimizing complex, non-natural active sites and expanding their functional diversity [83].

HTS platforms employ miniaturized reaction formats, microfluidic droplet systems, fluorescence-based readouts, and automated robotic pipetting to conduct millions of reactions per day with nanoliter-scale reagent consumption [84]. For example, droplet-based microreactors can screen up to 10^6^ synzyme variants per hour while simultaneously measuring activity via fluorescence output, enabling real-time functional profiling [85].

These systems are further enhanced by advances in directed evolution and combinatorial chemistry, which generate large variant libraries with controlled sequence diversity [86]. Through iterative screening-selection cycles, HTS enables the refinement of synzymes toward industrial goals, such as higher turnover rates, e.g., > 10^5^ M^−1^ s^−1^ reported for MOF-based peroxidase mimics and improved resilience against solvent exposure, extreme pH, and thermal fluctuation [13].

AI integration into HTS workflows allows automated analysis of high-dimensional assay data, facilitating early identification of promising synzyme candidates and reducing false positives. Machine learning models can learn from prior screening rounds to predict and prioritize the next generation of library members, thereby shortening development timelines [87].

Despite its transformative potential, HTS is not without limitations. The risk of false positives or negatives arising from assay variability and signal-to-noise inconsistencies can lead to inaccurate selection outcomes [88]. Moreover, low-activity variants often escape detection in threshold-dependent assays, and the high cost and complexity of setting up microfluidic HTS platforms remain substantial barriers to widespread adoption [89, 90].

In summary, HTS plays a critical role in accelerating synzyme discovery and refinement, particularly when integrated with AI-driven decision-making. Its ability to systematically test vast variant libraries makes it indispensable for next-generation enzyme design targeting industrial, biomedical, and environmental applications [91, 92].

## Biomedical Applications of Synzymes

Enzymes are central to physiological processes at both cellular and systemic levels, where they catalyze biochemical reactions critical for maintaining homeostasis [79]. In biomedicine, therapeutic enzymes have been employed for decades, with notable examples including monoamine oxidase inhibitors for neurological disorders and β-lactamase enzymes used in antibiotic manufacturing [93, 94]. Protein-based therapeutics, including recombinant enzymes such as asparaginase or tissue plasminogen activator, have demonstrated significant clinical utility in oncology and cardiovascular disease [95, 96].

However, the structural complexity, immunogenicity, and environmental sensitivity of natural enzymes have limited their broader therapeutic use, leading to increased interest in engineered and synthetic alternatives such as synzymes [97]. Synzymes are artificial constructs designed to mimic enzymatic activity, often exhibiting superior stability, substrate selectivity, or modularity compared to their natural counterparts [98, 99].

One of the earliest demonstrations of biomedical synzyme application came in 2000, when a DNA-aptamer conjugated to horseradish peroxidase (HRP) was engineered to mimic peroxidase activity and catalyze the oxidation of small molecules via hydrogen peroxide [100]. This synthetic DNAzyme system illustrated that artificial biocatalysts could perform oxidative transformations similar to those carried out by natural enzymes [101].

In 2003, *N*-methylmesoporphyrin IX, a synthetic heme analog, was incorporated into DNA frameworks, emulating the function of heme-containing peroxidases such as HRP and cytochrome P450s [102]. More recent studies by Tran and Ten demonstrated that engineering the binding domain between synthetic heme and DNA could produce synzymes with up to 230-fold increases in catalytic efficiency compared to baseline systems, approaching the performance of natural enzyme–substrate complexes [103].

Since 2020, there has been a notable surge in biomedical synzyme research, with over 60% of synzyme-related publications emerging in the last few years [104]. Applications include synthetic oxidases and peroxidases used in tumor microenvironment modulation, reactive oxygen species (ROS) scavenging, and site-specific drug activation. Ongoing efforts focus on developing biocompatible synzyme platforms suitable for in vivo use, with improved pharmacokinetics and minimal immunogenicity.

## Industrial Applications

### Food and Beverage Sector

#### The Role of Synthetic Enzymes in Fermentation Processes

Synthetic enzymes, or synzymes, have revolutionized fermentation processes in the food and beverage industry by enhancing efficiency and product quality [101]. In brewing, engineered amylases and glucanases break down complex carbohydrates into fermentable sugars, optimizing alcohol production and improving flavor profiles [105]. Proteases are utilized to modify protein content, influencing the texture and clarity of beverages [106]. The dairy industry benefits from synthetic rennin and lipases, which accelerate cheese ripening and develop distinct flavors. Overall, the integration of synzymes in fermentation processes leads to consistent product quality, reduced processing times, and enhanced sensory attributes [107].

#### Enhancement of Flavor and Aroma Profiles

Synzymes play a pivotal role in developing and enhancing flavor and aroma profiles in various food products [108]. In the production of fruit juices and wines, pectinases and cellulases break down cell walls, releasing aromatic compounds and increasing juice yield [109]. Lipases are employed to hydrolyze fats, generating free fatty acids that contribute to the characteristic flavors in cheeses and other dairy products [110]. Additionally, engineered glycosidases release bound aromatic compounds in wines, intensifying their bouquet and complexity. The precise application of these synzymes allows manufacturers to tailor flavor profiles to meet consumer preferences, enhancing the overall sensory experience of food and beverages [111].

#### Improvement of Shelf Life and Food Safety

Synzymes are increasingly utilized to enhance the shelf life and microbial safety of food products. Oxidoreductases, such as laccases, help prevent spoilage by removing phenolic compounds that contribute to off-flavors and oxidative degradation in juices and wines [112]. Antimicrobial enzymes, including lysozymes, are incorporated into food preservation systems to inhibit spoilage microorganisms and foodborne pathogens [113]. Unlike drug-targeted systems, synzymes in food applications act externally to block microbial growth or chemical degradation, rather than mediating internal therapeutic effects. These enzyme-based strategies reduce food waste, maintain quality during storage and transport, and improve consumer safety and confidence [114].

### Environmental Biotechnology

#### Synzymes in Wastewater Treatment and Biodegradation

Synzymes have emerged as effective agents in the treatment of wastewater and the biodegradation of environmental pollutants [21]. Engineered oxidases and peroxidases are employed to break down complex organic pollutants, including dyes and pharmaceuticals, into less harmful compounds, facilitating their removal from wastewater [115]. Laccases, in particular, have been applied to degrade a variety of environmental contaminants due to their broad substrate specificity. The use of synzymes in these processes enhances the efficiency of pollutant removal, contributing to cleaner water bodies and reduced environmental impact [116].

#### Role of Synzymes in Plastic Waste Management

Synzymes have been developed to address the growing concern of plastic pollution by enabling the biodegradation of synthetic polymers [21]. Enzymes such as PETases and cutinases have been engineered to break down polyethylene terephthalate (PET) and other plastics into their monomeric components, which can then be repurposed to produce new materials [117, 118]. This enzymatic recycling process offers a promising solution to reduce plastic waste accumulation and promote a circular economy. Advancements in this field aim to enhance the efficiency and specificity of synzymes, making large-scale plastic waste management more feasible and environmentally friendly [62].

### Biomedical Applications

#### Synzymes Can Be Utilized for Targeted Drug Release

Synzymes have gained significant attention in drug delivery systems due to their ability to catalyze specific biochemical reactions, making them ideal for targeted drug release [6]. Enzyme-responsive carriers selectively release therapeutics at disease sites where enzymatic activities are elevated. For instance, synzymes engineered to recognize cancer-specific proteases such as MMPs or cathepsins enable controlled release of chemotherapeutics at tumor sites, minimizing systemic toxicity [119].

Additionally, synzyme-functionalized nanoparticles respond to biological cues such as pH shifts, oxidative stress, or specific biomolecules—ensuring drug activation under precise physiological conditions and improving drug bioavailability, patient compliance, and therapeutic efficacy [120].

In gene therapy, synzymes enable site-specific cleavage of nucleic acids, allowing control over gene activation or suppression. CRISPR-associated synthetic enzymes, for example, have been used to correct disease-causing mutations with high precision [121]. Overall, synzymes represent a versatile and programmable platform for therapeutic innovation, with applications in oncology, infectious disease, and personalized medicine.

#### Synzymes in Biosensors for Disease Diagnosis

Synzymes are central to the advancement of biosensor technology by enhancing the sensitivity, specificity, and rapidity of disease detection. They can be designed to recognize key biomarkers such as glucose (diabetes), lactate (metabolic stress), or tumor-associated antigens (oncology), triggering a detectable signal via colorimetric, electrochemical, or fluorescent responses [122, 123].

For example, glucose oxidase-mimicking synzymes are integrated into commercial blood glucose meters, enabling real-time monitoring for diabetic patients. In infectious disease diagnostics, peroxidase-like synzymes generate visible or electrochemical signals in response to viral or bacterial targets (e.g., COVID-19, TB, HIV) [124].

As discussed in previous sections, synzyme-functionalized nanoparticles can respond to internal biological stimuli. However, in biosensor applications, these mechanisms are directed toward signal generation rather than therapeutic action, particularly in wearable sensors and lab-on-a-chip devices supporting point-of-care and real-time diagnostics [125].

## Future Perspectives and Research Directions

The future of synzyme research is poised for transformative advancements through interdisciplinary collaboration across computational chemistry, nanotechnology, and molecular biology. One of the most promising avenues is the application of AI and machine learning in enzyme design. Computational modeling is expected to play a critical role in predicting catalytic efficiencies, optimizing active site architectures, and enhancing substrate specificity. AI-driven approaches will accelerate the discovery of novel synzymes with superior performance in industrial and biomedical applications.

The development of hybrid enzymes that integrate synthetic catalysts with natural enzyme components is another area of interest. These hybrid systems aim to combine the high efficiency of natural enzymes with the enhanced stability and tunability of synthetic molecules. Such advancements could lead to breakthroughs in biomimetic catalysis, allowing synzymes to function more effectively within complex biological environments.

Regenerative medicine is also expected to benefit from synzyme innovation. Researchers are exploring the use of synthetic enzymes to promote tissue regeneration, facilitate wound healing, and develop bioengineered organs. Synzymes could be used to create self-healing biomaterials that respond dynamically to physiological changes, paving the way for next-generation therapeutic solutions.

In the realm of personalized medicine and nanomedicine, synzymes hold great potential for targeted drug delivery and controlled biochemical reactions at the cellular level. By tailoring synzyme activity to individual patient profiles, researchers could achieve highly selective therapies that minimize side effects while maximizing therapeutic efficacy.

Sustainability and green chemistry approaches will also shape the future of synzyme development. The focus will be on designing environmentally friendly catalytic processes that reduce chemical waste and energy consumption. Innovations in enzyme engineering may lead to synzymes that facilitate carbon capture, pollutant degradation, and bioremediation, contributing to global sustainability efforts.

**Fig. 5:**
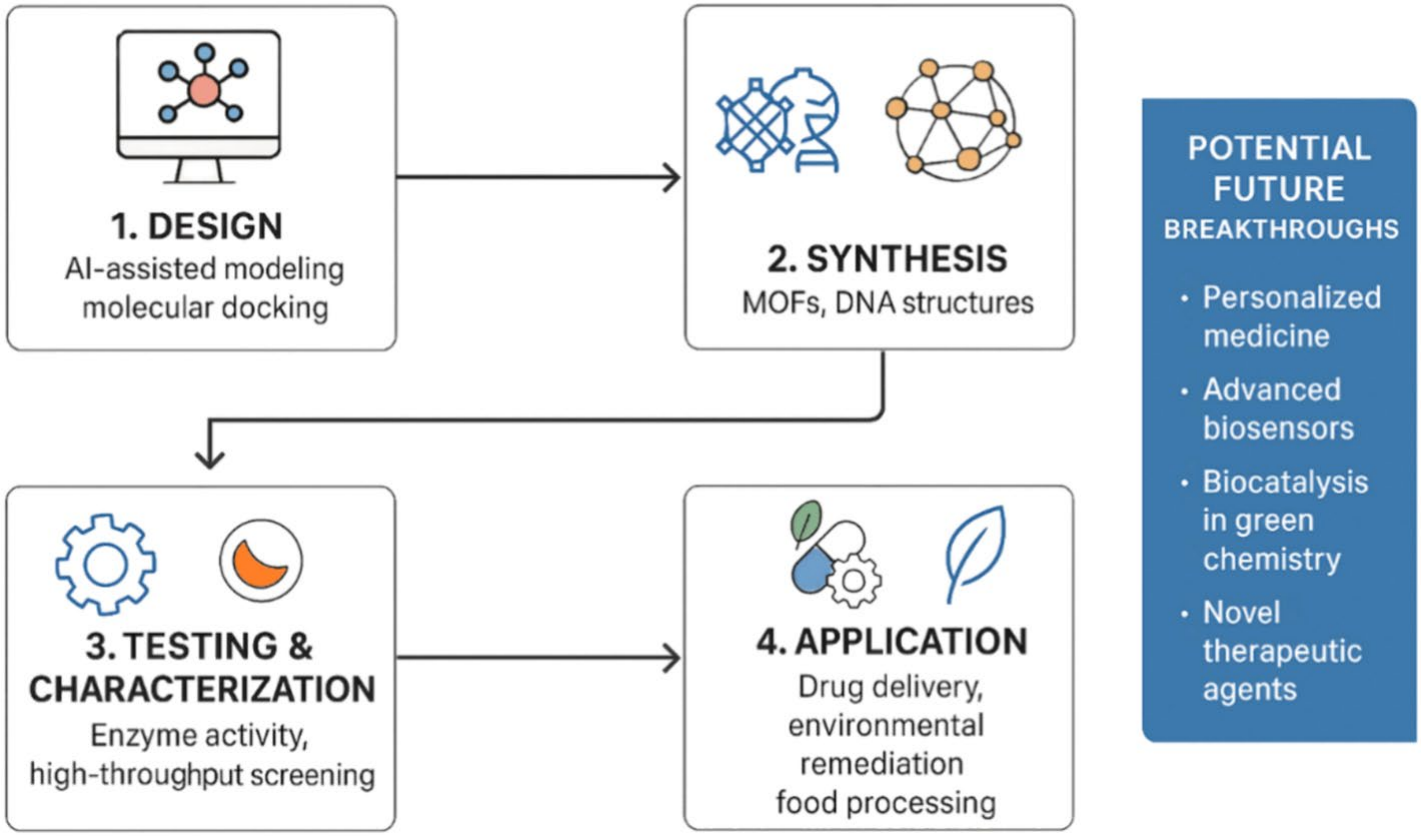
Synzyme engineering pipeline and projected innovation domains

Overall, the continued integration of computational advancements, biomimetic engineering, and sustainable chemistry will drive the next generation of synzyme technologies. By addressing current limitations and expanding functional capabilities, synzymes are poised to revolutionize multiple scientific and industrial domains in the coming years. Projected future breakthroughs in synzyme engineering are illustrated in (Fig. 5), highlighting emerging trends in smart biosensors, personalized therapy, and green biocatalysis.

This pipeline diagram highlights key stages in synzyme engineering—from AI-assisted design to real-world applications—and features a side banner outlining projected innovation areas such as personalized medicine, advanced biosensors, green biocatalysis, and novel therapeutic agents. The figure reflects the interdisciplinary evolution of synzyme technology. Abbreviations: AI, artificial intelligence; MOF, metal–organic framework. Created by the author for illustrative purposes.

## Conclusion

Synzymes have emerged as a compelling alternative to natural enzymes, offering remarkable advantages in catalytic stability, tunability, and substrate specificity across biomedical, industrial, and environmental domains. Their ability to function under extreme pH, temperature, or solvent conditions has positioned them as valuable tools in areas where traditional biocatalysts often fall short. Recent advances in nanotechnology, supramolecular chemistry, and computational enzyme design have accelerated the development of highly efficient synzyme systems, enabling their integration into diverse applications such as targeted therapy, pollutant degradation, and green manufacturing.

However, despite their transformative potential, synzymes face important limitations that must be addressed before their widespread adoption. Scalable synthesis remains challenging, often involving costly or complex procedures that hinder commercial viability. In biomedical contexts, concerns related to immunogenicity, toxicity, and long-term biostability remain largely unresolved. Moreover, many synthetic enzymes still lack the catalytic efficiency, adaptability, and self-regulatory mechanisms found in natural systems. Regulatory frameworks for synzyme deployment, especially in therapeutic or environmental settings, are also underdeveloped and demand clearer standardization.

Future research must therefore move beyond proof-of-concept demonstrations and focus on improving biocompatibility, substrate range, and responsiveness to dynamic environments. The continued refinement of AI and high-throughput screening will be instrumental in accelerating synzyme optimization, while interdisciplinary collaboration will be essential to overcome remaining biochemical and engineering challenges. As these barriers are gradually addressed, synzymes are expected to play an increasingly central role in precision medicine, sustainable catalysis, and next-generation biotechnology, shaping the future of synthetic biocatalysis and enzyme innovation.

## Data Availability

No datasets were generated or analyzed during the current study.

## Declarations

### Use of Artificial Intelligence Tools

AI tools, including ChatGPT by OpenAI, were used in the preparation of this manuscript to assist in language editing, formatting, and generating structured sections. The content, ideas, and conclusions presented are solely the work of the author.
